# Bioinformatic search of plant microtubule-and cell cycle related serine-threonine protein kinases

**DOI:** 10.1186/1471-2164-11-S1-S14

**Published:** 2010-02-10

**Authors:** Pavel A Karpov, Elena S Nadezhdina, Alla I Yemets, Vadym G Matusov, Alexey Yu Nyporko, Nadezhda Yu Shashina, Yaroslav B Blume

**Affiliations:** 1 Institute of Food Biotechnology and Genomics, National Academy of Sciences of Ukraine, 04123 Kyiv, Ukraine; 2 Institute of Protein Research, Russian Academy of Sciences, 142290 Pushchino, Moscow Region, Russian Federation; 3 AN Belozersky Institute of Physical-Chemical Biology, Moscow State University, Leninsky Gory, 119992 Moscow, Russian Federation

## Abstract

A bioinformatic search was carried for plant homologues of human serine-threonine protein kinases involved in regulation of cell division and microtubule protein phosphorylation (SLK, PAK6, PAK7, MARK1, MAST2, TTBK1, TTBK2, AURKA, PLK1, PLK4 and PASK). A number of SLK, MAST2 and AURKA plant homologues were identified. The closest identified homologue of human AURKA kinase was a protein of unknown function, A7PY12/GSVIVT00026259001 from *Vitis vinifera *(herein named as "STALK", Serine-Threonine Aurora-Like Kinase). Analysis of STALK's three-dimensional structure confirmed its relationship to the subgroup of AURKA-like protein kinases.

## Background

Microtubules are essential and universal cytoskeleton component of all eukaryotic cells [1-3]. Due to their dynamic behaviour, microtubules participate in many cellular processes, including cell division, shape formation, intracellular trafficking and organelle positioning. Their protofilaments are composed of tubulin heterodimers, a highly conserved microtubule protein [4,5]. Their multiple isotypes and isoforms arise from variable gene expression as well as post-translational modifications, most studied of which are phosphorylation, acetylation, tyrosination/detyrosination, polyglutamylation, polyglycylation, and palmitoylation [3,6,7]. Among these modifications, phosphorylation represents a special case because of its wide occurrence [8] and ability to regulate structure/function of around 30% of all proteins in eukaryotes [9,10].

The genome of *Arabidopsis thaliana *contains more then 1000 protein kinase genes [8]. Analysis of plant gene similarity to the phosphate-binding regions of animal tyrosine kinases showed that genes for Zap70 tyrosine kinase family are the most similar to its potential plant homologues [11,12]. tBLASTn http://blast.ncbi.nlm.nih.gov/ scanning of *Arabidopsis *genome against mouse Zap70 catalytic domain detected 503 consensus regions corresponding to 494 protein kinases [12]. It means that only ~50% of *Arabidopsis *kinases are homologous to animal kinases. These genes are distributed between different chromosomes: 150 - on I, 71 - on II, 94 - on III, 65 - on IV and 114 - on chromosome V. Analysis in SMART http://smart.embl-heidelberg.de/ confirms the correspondence of their products to the models of catalytic domains of S_TKc, STYKc, TyrKc, Pkinase, Pkinase_Tyr, S_TKc, D1phk (SCOP: 88854), D1pme (SCOP: 88854), D1qpca (SCOP: 88854), D1fgka (SCOP: 88854), D1ir3a (SCOP: 88854), D1b6cb (SCOP: 88854), 1A06, 1BYG, 1CM8, 1E1V, 1F3M, 1FGI, 1FOT, 1FPU, 1FVR, 1G3N, 1GOL, 1IA8, 1IAN, 1JPA, 1KOA, 1PME, 1QCF, 1QL6, 1QPD, 2PTK, 2SRC, 3LCK and d1b6cb kinases [12]. We previously demonstrated that microtubule tubulin in plants, like in animals, is highly phosphorylated by different serine/threonine [13] and tyrosine kinases [14,15]. It is interesting that among potential products of the genes we identified proteins corresponding to HMMs and patterns of catalytic domains of atypical Tyr-specific kinases (Pkinase_Tyr), dual specific (STYKc) and canonical animal tyrosine kinases (TyrKc) [11,12].

Phosphorylation of other proteins forming plant microtubules and involved in cell division, may also be mediated by kinases having well characterized homologues in the animal kingdom [15]. Several microtubule proteins show homology between animals and plants, e.g., microtubule-associated proteins type I (MAP1) [16]. Therefore, these proteins may be expected to have similar phosphorylation sites and to be phosphorylated by corresponding conserved protein kinases.

Microtubule-related proteins are both species- and tissue-specific. Not surprisingly, these proteins differ significantly between plants and animals [16-18]. Accordingly, the total microtubule-related kinase subset (termed "kinome") is distinct in animals and plants. However, microtubule proteins and associated kinases in plant cells have not been studied in as much details as in their human/animal counterparts. Further insight requires identifying of plant kinases and corresponding genes.

All protein kinases are characterized by a conserved catalytic (kinase) domain of 250-300 residues which, in turn, contains unique sub-domain motifs related to serine/threonine, tyrosine or dual kinases [19,20]. These features enable search and identification by database scanning using BLAST family tools [21]. It is this catalytic domain with the characteristic structure/function relationship that aids in identification of new kinases by *in silico *methods. The homology of these domains has been previously used to identify several protein kinases from multicellular organisms [22-27].

At present, several eukaryotic genomes have been completely sequenced and partially annotated. Specific examples of plant genomic information include plant genomic data in NCBI http://www.ncbi.nlm.nih.gov/, and, additionally, the project Genoscope http://www.genoscope.cns.fr which provides information and *in silico *search engines for the following plants: *Arabidopsis thaliana, Ectocarpus siliculosus, Medicago truncatula, Oryza sativa, Alnus glutinosa, Aphanomyces euteiches, Casuarina glauca, Citrus clementina, Eucalyptus, Juglans regia, Phaseolus vulgaris, Pinus pinaster, Populus trichocarpa × deltoides, Quercus, Saccharum *ssp., *Theobrama cacao, Triticum *ssp.*, Vitis vinifera.*

The goal of the present work was to identify plant homologues of human kinases [28] that could participate in phosphorylation of microtubule proteins and/or regulation of cell division. The investigated kinase families included AGC (containing kinases A, G and C families), CAMK (calcium/calmodulin-dependent), CK1 (casein kinase 1) and STE (homologues of yeast Sterile 7, Sterile 11 and Sterile 20 kinases) protein kinases. For the bioinformatic search and reconstruction of plant microtubule kinome, we used sequences of the following human protein kinases: SLK, PAK6, PAK7 (from the superfamily STE); MARK1, PASK (from the superfamily CAMK); MAST2, AURKA (from the superfamily AGC); TTBK1, TTBK2 (from the superfamily CK1); kinases Plk1 and Plk4 (not belonging to any specific group) [28].

## Results and discussion

**Figure 1:**
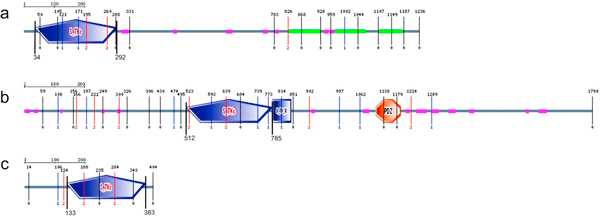
**SLK_HUMAN (a), MAST_HUMAN (b) and STK6_HUMAN (c) kinase domain architecture**. S_TKc - catalytic domain of serine/threonine-kinases; S_TK_X - auxiliary S_TKc domain; DUF1908 - domain of unknown function (DUF1908); PDZ (also referred as DHR (Dlg homology region) or GLGF (relatively well conserved tetrapeptide in these domains) - domain found in PSD-95, Dlg and ZO-1/2[74]. These domains help anchor transmembrane proteins to the cytoskeleton and hold together signaling complexes [72].

Using public databases and published research papers [28-47], we have selected a range of human kinases involved in microtubule proteins phosphorylation and cell division regulation (Table 1). Depending on the complexity level of protein kinases domain organization, either full sequences (in case of AURKA) or catalytic domain sequence (SLK, PAK7, MARK1, SLK, MAST2 TTBK1, PLK1, PLK4 and PASK, as seen on Figure 1) were used for SIB BLASTp scanning. As a result of the UniProt database scanning, we identified plant homologues of human kinases from the families SLK, MAST2 and AURKA.

**Table 1:** Human kinases taking part in microtubule protein phosphorylation and cell division regulation [28-30]; in italics are kinases plant homologues of which were identified in the present study

Protein kinase	GenBank (mRNA)	Gene	Locus	UniProt
*SLK (KIAA0204, LOSK, MGC133067, STK2, bA16H23.1, se20-9) *[32,33,47]	NM_014720	NC_000010.10	*Chr.10; Loc.10q25.1*	*Q9H2G2*
PAK7 (RP5-1119D9.3, KIAA1264, MGC26232, PAK5) [35,39]	NM_177990	NC_000020.10	Chr.20; Loc.20p12	Q9P286
PAK6 (PAK5) [35,39]	NM_020168	NC_000015.9	Chr.15; Loc.15q14	Q9NQU5
MARK1 (KIAA1477, MARK, MGC126512, MGC126513) [34]	NM_018650	NC_000001.10	Chr.1; Loc.1q41	Q9P0L2
*MAST2 (MTSSK, MAST205, FLJ39200, KIAA0807, RP4-533D7.1) *[45]	NM_015112	NC_000001.10	*Chr.1; Loc.1p34.1*	*Q6P0Q8*
TTBK1 (BDTK, KIAA1855, RP3-330M21.4) [42]	NM_032538	NC_000006.11	Chr.6; Loc.6p21.1	Q5TCY1
TTBK2 (KIAA0847, SCA11, TTBK) [37]	NM_173500	NC_000015.9	Chr.15; Loc.15q15.2	Q6IQ55
*AURKA (AIK, ARK1, AURA, AURORA2, BTAK, MGC34538, STK15, STK6, STK7) *[38,41,43,46]	NM_198436	NC_000020.10	*Chr.20; Loc.20q13.2-q13.3*	*O14965*
PLK1 (PLK, STPK13) [40,41,43,44]	NM_005030	NC_000016.9	Chr.16; Loc.16p12.1	P53350
PLK4 (SAK, STK18) [31,41]	NM_014264	NC_000004.11	Chr.4; Loc.4q28	O00444
PASK (DKFZp434O051, DKFZp686P2031, KIAA0135, PASKIN, STK37) [36]	NM_015148	NC_000002.11	Chr.2; Loc.2q37.3	Q96RG2

### Plant homologues of SLK_HUMAN (Q9H2G2)

**Figure 2:**
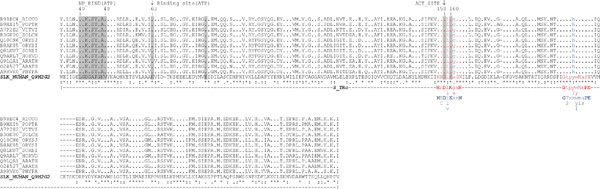
**Catalytic domain alignment of human SLK_HUMAN (Q9H2G2) and putative plant SLK-like proteins**. **NP_BIND (ATP) - nucleotide phosphate binding region**; Binding site (ATP) - ATP binding site; ACT_SITE - proton acceptor; consensus conserved motif in catalytic loop region of the subdomain VIb (animals (red): H-R-D-[LI]-K-[GA]-x-N and *A. thaliana *(blue): H-[RC]-D-[ILV]-K-x-x-N); consensus conserved motif in activation loop of the subdomain VIII (in animals (red): G-T-P-[YF]-[WY]-M-A-P-E and in *A. thaliana *(blue): G-[TS]-x-x-[WYF]-[ML]-[AS]-P-E)

Uniprot scanning against SLK_HUMAN catalytic domain has revealed 11 plant homologues to STE20-like human kinase (Table 2) from *A. thaliana *(Q9LQA1/AT1G69210, O24527/At1g69220), *Hordeum vulgare *var. *distichum *(Q9ARL7/GenBank: AY013246.1), *O. sativa *ssp. *japonica *(Q10CN6/Os03g0755000), *O. sativa *ssp. *indica *(B8AK85/OsI_13553), *Physcomitrella patens *ssp. *patens *(A9RVK0/PHYPADRAFT_119967), *Populus balsamifera *ssp. *trichocarpa *(B9HXI5/POPTRDRAFT_226120), *Ricinus communis *(B9REC4/RCOM_1620250), *Solanum chacoense *(B3GK00/MAP4K1) [48 Champion et al., 2004a], *Sorghum bicolor *(Q8LKU7/Sb01g007720) and *V. vinifera *(A7P2E2/GSVIVT00030023001). Consensus region identity to *Homo sapiens *kinase SLK (Q9H2G2) catalytic region reached 45-46% with similarity of 65-66% (Table 2, Figure 2). Analysis of full sequences of potential plant homologues in SMART confirmed their relationship to serine/threonine-specific protein kinases. All identified plant homologues were found in TrEMBL database as deposited proteins with unknown function. As an exception, putative STE20 protein O24527/At1g69220 (TrEMBL database), from *A. thaliana*, is annotated as potential homologue of protein kinase SIK1.

**Table 2:** Plant homologues for animal kinases taking part in microtubule protein phosphorylation and cell division regulation

Protein kinase from Homo sapiens	Potential plant homologues	Species	Database	Length of consensus	Score	Expect (E-value)	Identity (%)	Similarity (%)	Gaps (%)
SLK	B9REC4_RICCO	*Ricinus communis*	tr	260	245	3e-63	46	66	2
	A9RVK0_PHYPA	*Physcomitrella patens *ssp. *patens*	tr	260	243	1e-62	46	65	2
	A7P2E2_VITVI	*Vitis vinifera*	tr	260	243	1e-62	46	65	2
	B9HXI5_POPTR	*Populus balsamifera *ssp. *trichocarpa*	tr	261	239	2e-61	46	65	2
	Q9LQA1_ARATH	*A. thaliana*	tr	260	242	2e-62	46	66	2
	O24527_ARATH	*-*	tr	260	242	2e-62	46	66	2
	Q10CN6_ORYSJ	*Oryza sativa *ssp. *japonica*	tr	260	241	4e-62	45	65	2
	B8AK85_ORYSI	*Oryza sativa *ssp. *indica*	tr	260	241	4e-62	45	65	2
	Q8LKU7_SORBI	*Sorghum bicolor*	tr	260	241	5e-62	45	65	2
	Q9ARL7_HORVD	*Hordeum vulgare *var. *distichum*	tr	260	239	1e-61	45	65	2
	B3GK00_SOLCH	*Solanum chacoense*	tr	260	238	3e-61	45	66	2

MAST2	A7QWR7_VITVI	*Vitis vinifera*	tr	359	314	5e-83	47	63	4
	A7PHB5_VITVI*	-	tr	346	313	7e-83	46	64	1
	A7NTE9_VITVI*	-	tr	327	295	3e-77	46	63	4
	A5BWH0_VITVI*	-	tr	338	241	4e-61	39	56	9
	
	Q9LE81_ARATH	*A. thaliana*	tr	350	310	9e-82	46	63	4
	Q94F38_ARATH*	-	tr	343	301	4e-79	46	62	3
	Q8GZ40_ARATH*	-	tr	360	300	6e-79	44	63	4
	Q0WLU7_ARATH	-	tr	343	294	4e-77	45	63	4
	
	Q10E10_ORYSJ	*O. sativa *ssp. *japonica*	tr	384	309	2e-81	44	58	7
	Q2QM12_ORYSJ	-	tr	359	305	3e-80	46	61	6
	
	A2XLA4_ORYSI	*O. sativa *ssp.*indica*	tr	384	309	2e-81	44	58	7
	A2ZMW0_ORYSI	-	tr	359	279	2e-72	44	59	9
	
	Q32YB5_MEDTR	*Medicago truncatula*	tr	364	307	6e-81	45	62	6
	
	A9TQ65_PHYPA*	*Physcomitrella patens *ssp. *patens*	tr	347	286	1e-74	43	60	5
	A9TWY7_PHYPA	-	tr	327	285	2e-74	43	61	6
	A9TUB0_PHYPA*	-	tr	326	285	3e-74	44	61	5
	A9T694_PHYPA*	-	tr	364	284	4e-74	42	56	9

AURKA	Q5SNH4_ORYSJ	*O. sativa *ssp. *japonica*	tr	285	369	e-100	64	77	2
	Q4R1K7_ORYSJ	-	tr	264	359	2e-97	66	80	1
	
	A2WLL4_ORYSI	*O. sativa *ssp.*indica*	tr	285	369	e-100	64	77	2
	
	A9PFI9_POPTR	*Populus balsamifera *ssp. *trichocarpa*	tr	285	368	e-100	62	78	1
	
	A7P4F7_VITVI	*V. vinifera*	tr	285	368	e-100	63	77	1
	A5BPE0_VITVI	-	tr	283	367	e-100	63	77	1
	A7PY12_VITVI	-	tr	279	339	3e-91	56	76	1
	
	B4F8A1_MAIZE	*Zea mays*	tr	292	368	e-100	62	76	2
	
	AUR2_ARATH	*A. thaliana*	sp	272	365	3e-99	65	78	1
	AUR2_ARATH sof.2	-	sp	268	365	4e-99	65	79	1
	AUR1_ARATH	-	sp	284	363	2e-98	60	76	1
	AUR3_ARATH	-	sp	280	337	1e-90	57	76	1

* - presence of auxiliary catalytic S_TK_X domain

**Figure 3:**
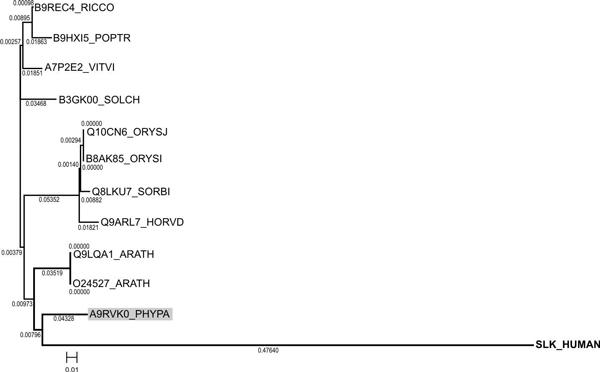
**Phylogenetic tree constructed for SLK kinase of *H. sapiens *and its plant homologues**.

To identify the closest homologues among the potential plant SLK-like kinases, we performed multiple alignments of catalytic domains with that of human SLK and built a phylogenetic tree using the neighbour joining method. According to the cladistic data, a common clade with human SLK is formed by proteins Q9LQA1/At1g69210 and O24527/At1g69220 from *A. thaliana *as well as A9RVK0 from *P. patens *ssp. *patens *(Figure 3). Following that, the closest plant homologue of human SLK was A9RVK0 from *P. patens *ssp. *patens *with the identity of 46% and similarity of 65%. Inside the group, the newly found plant homologues demonstrated a high degree of sequence identity with conserved functionally important positions specific to SLK kinases. Also, the analysis of the known conserved residues, implicated in substrate recognition in animal and *A. thaliana *STE-kinases [49 Champion et al., 2004b], confirmed the existence of the consensus motifs in catalytic loop region of the subdomain VIb (animals: H-R-D-[LI]-K-[GA]-x-N and *A. thaliana*: H-[RC]-D-[ILV]-K-x-x-N) and activation loop of the subdomain VIII (animals: G-T-P-[YF]-[WY]-M-A-P-E and *A.thaliana*: G-[TS]-x-x-[WYF]-[ML]-[AS]-P-E) in all identified plant homologues(Figure 2).

### Plant homologues of MAST2_HUMAN (Q6P0Q8)

As with SLK above, we searched the UniProt database for potential plant homologues of MAST2_HUMAN with the catalytic domain sequence identified in SMART tool http://smart.embl-heidelberg.de/[50].

**Figure 4:**
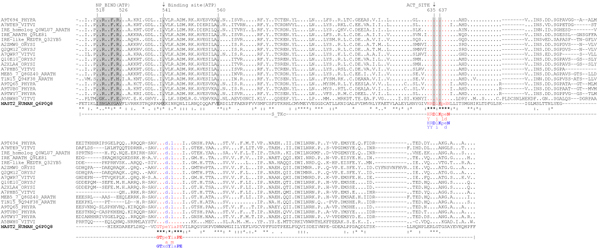
**Catalytic domain alignment of MAST_HUMAN (Q6P0Q8) with its plant homologues**. **NP_BIND (ATP) - nucleotide phosphate binding region**; Binding site (ATP) - ATP binding site; ACT_SITE - proton acceptor; consensus conserved for AGC kinases motif in catalytic loop region of the subdomain VIb (animals (red): [HY]-R-D-[LI]-K-[PL]-[ED]-N and *A. thaliana *(blue): [HY]-[RY]-D-[LI]-K-P-[ED]-N); consensus conserved for AGC kinases motif in activation loop of the subdomain VIII (in animals (red): G-T-P-[EA]-Y-[IM]-A-P-E and in *A. thaliana *(blue): G-T-x-D-Y-L-A-P-E)

Swiss-Prot/TrEMBL scanning has revealed 17 plant MAST2_HUMAN homologues: 4 from *V. vinifera*, 4 from *A. thaliana*, 2 from *O. sativa *ssp. *japonica*, 2 from *O. sativa *ssp. *indica*, 1 from *Medicago truncatula *and 4 from *P. patens *ssp. *patens *(Table 2). Animal MAST2 kinases contain a typical auxiliary C-terminal S_TK_X domain (sTKc or sTKx), termed C-terminal kinase domain [51], which, together with the catalytic domain, is responsible for interactions with microtubules [45]. Results of the domain architecture analysis of full sequences of potential plant homologues of human MAST2 kinase demonstrated that only 8 out of 17 found sequences have the C-terminal S_TK_X domain (SMART SM00133): A7PHB5/GSVIVT00017880001, A7NTE9/GSVIVT00014640001, A5BWH0/VITISV_001730 (all from *V. vinifera*); Q94F38/At1g48490, Q8GZ40/At3g17850 (*A. thaliana*) and A9TQ65/PHYPADRAFT_148894, A9TUB0/PHYPADRAFT_150879, A9T694/PHYPADRAFT_140919 (*P. patens *ssp. *patens*) (Table 2). Consequently, the maximum consensus identity for the query catalytic domain in BLASTp was observed with a protein of unknown function, A7QWR7/GSVIVT00008775001, from grape (identity-47%, similarity-63%) (Figure 4, Table 2). Also, the analysis of the known conserved residues, implicated in substrate recognition in animal and *A. thaliana *AGC-kinases[49]., confirmed the existence of the consensus motifs in catalytic loop region of the subdomain VIb (animals: [HY]-R-D-[LI]-K-[PL]-[ED]-N and *A. thaliana*: [HY]-[RY]-D-[LI]-K-P-[ED]-N) and activation loop of the subdomain VIII (animals: G-T-P-[EA]-Y-[IM]-A-P-E and *A.thaliana*: G-T-x-D-Y-L-A-P-E) in all identified plant MAST-homologues(Figure 2).

**Figure 5:**
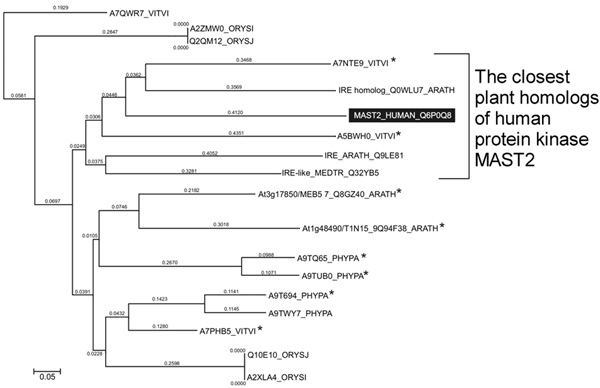
Phylogenetic tree built for *H. sapiens *MAST2 kinase and its plant homologues; * - plant homologues containing typical for animal MAST2 kinases auxiliary S_TK_X catalytic S_TK_X domain.

Phylogenetic analysis using Neighbour-Joining algorithm based on catalytic domain similarity demonstrated that a common clade with human MAST2 is formed by the following plant homologues: Q0WLU7/At1g45160, Q9LE81/At5g62310 (*A. thaliana*), A7NTE9_VITVI*/GSVIVT00014640001, A5BWH0_VITVI*/VITISV_001730 (*V. vinifera*) and Q32YB5/GenBank: AY770392.1 (*M. truncatula*) (Figure 5).

**Figure 6:**
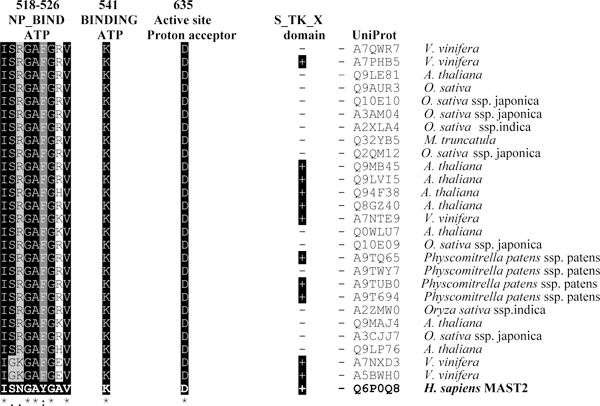
**Similarity of *H. sapiens *MAST2 and potential plant homologues for auxiliary S_TK_X domain, conserved active site Asp-635 and ATP-binding motifs (NP_BIND ATP and Lys-541)**.

Therefore, the most distinguished plant homologues of the MAST2 family are A7NTE9_VITVI*/GSVIVT00014640001 (423 residues; chromosome 18), A5BWH0_VITVI*/VITISV_001730 (550 residues; chromosome 5) from *V. vinifera*. In summary, based on the analysis of human MAST2 and its potential plant homologues for such functional elements as auxiliary S_TK_X domain, Asn635 in putative active centre, ATP-binding motif (NP_BIND ATP) and Lys541, allowed us to conclude that A7NTE9 (ORF Name GSVIVT00014640001) from grape (Figure 6) is the most promising for future research including spatial structure reconstruction and molecular genetic methods.

### Plant homologues of human Aurora A kinase (STK6_HUMAN, O14965)

Protein kinase Aurora A belongs to the serine/threonine kinase family AGC [28] and plays a fundamental role in mitosis regulation [30,31]. In the case of Aurora A kinase, we used the full sequence of human STK6 (AURKA; O14965) as a 'query' sequence for the BLASTp search. As a result of database scanning, we have identified 12 potential plant homologues of the STK6 kinase (Table 2) characterized by a similar domain structure and high sequence similarity of kinase domains and N- and C-terminals. Sequence similarity has reached 76-80% with the identity of 56-66%.(Table 2)

**Figure 7:**
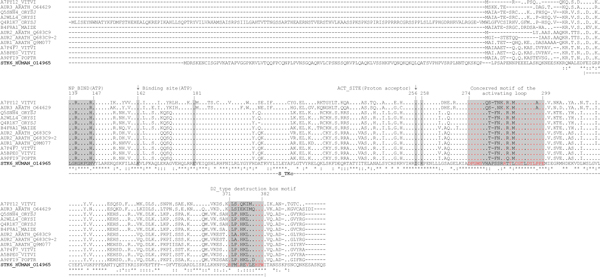
**Alignment of human Aurora A and its plant homologues**. NP_BIND (ATP) - nucleotide phosphate binding site; Binding site (ATP) - ATP binding site; ACT_SITE - proton acceptor site

In an earlier paper on plant homologues of Aurora kinases [52], the authors described a high level of identity for Aurora catalytic domains from *Arabidopsis *(64-95%). We now report additional homologues belonging to *O. sativa *ssp. *japonica *(Q5SNH4/Os01g0191800, Q4R1K7/OsAUR1), *O. sativa *ssp. *indica *(A2WLL4/OsI_00731), *P. balsamifera *ssp. *trichocarpa *(A9PFI9/POPTRDRAFT_819526), *V*. *vinifera *(A7P4F7/GSVIVT00032134001, A5BPE0/VITISV_023325, A7PY12/GSVIVT00026259001), *Zea mays *(B4F8A1/LOC100191291) and *A. thaliana *(AUR2 Isof. 1 and AUR2 Isof. 2 (At2g25880), AUR1/At4g32830, AUR3/At2g45490). Together with a low gap percentage (1-2%), such a high identity reflects not only sequence homology but also homology of structure and function (Figure 7). As evident from the annotations in the Swiss-Prot database kinases AUR1/At4g32830, AUR2 Isof. 1 and AUR2 Isof. 2 (At2g25880) and AUR3/At2g45490 from *A. thaliana *could be functional homologues. Sequence Q4R1K7/OsAUR1 from *O. sativa *ssp. *japonica *in the TrEMBL database [53] is also annotated as a potential plant Aurora kinase.

**Figure 8:**
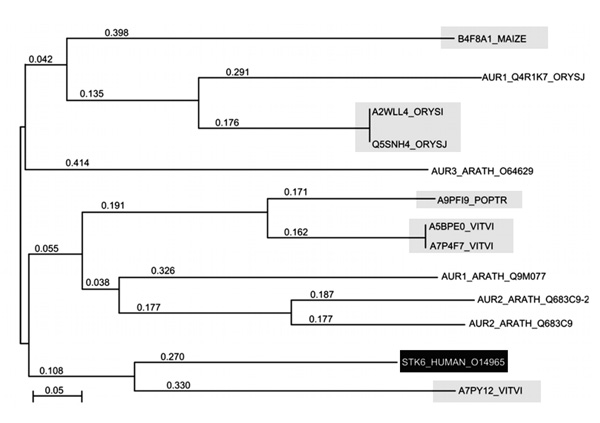
**Phylogenetic tree of STK6_HUMAN (Aurora A) kinase and its plant homologues demonstrating their phylogenetic relationships**. Highlighted are the proteins identified in the paper.

As a result of highly conserved positions of the functionally important residues and motifs, together with homology of a primary sequence and domains (analysis using domain profiles and hidden Markov models) all S_TKc kinases were identified as plant structural and functional homologues of human Aurora A. Based on the cladistic analysis (Figure 8), the closest homologue of STK6_HUMAN was a protein of unknown function, A7PY12/GSVIVT00026259001 from *V. vinifera *(identity-76%, similarity-56%, E-value = 3e-91).

**Figure 9:**
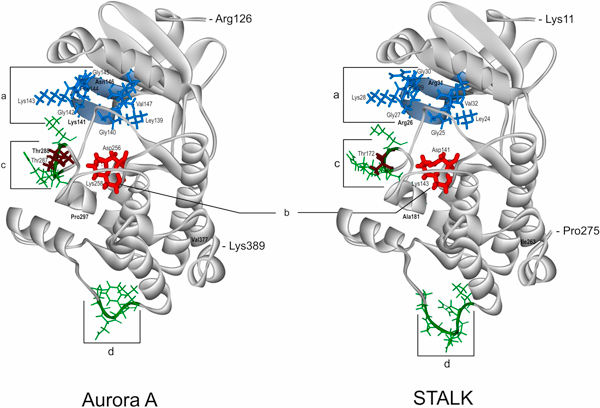
**Comparison of the catalytic domain spatial structures of the human protein kinase Aurora A (AURKA, STK6, PDB: **2J4Z**) and the protein of unknown function STALK (S_T AURKA LIKE KINASE, UniProt: A7PY12) from *V. vinifera***. "a" (marked by blue) ATP-binding regions in Aurora A and STALK; "b" (marked by red) is active site; "c", "d" (marked by green) are the most spatially variable regions between the two proteins; phosphorylated Thr residues (287, 288) in the Aurora A are marked by brown. In bold are marked the only discrepancies between the corresponding functionally important residues in Aurora A versus STALK: Asn146↔Arg31, Lys141↔Arg26 in "a"; Thr288↔Thr172 in variable region "c"; Pro297↔Ala181 in the DFGWSxxxxxxxRxTxCGTxDYLPPE motif of the activating loop; Val377↔Ile263 in the D2_type destruction box - Rxx(L/I)xxVxxHPW

Taking into account the critical role of Aurora kinases in microtubule protein [46] and cell division regulation [30,41,53], the plant homologue A7PY12/GSVIVT00026259001 from *V. vinifera*, referred herein as STALK (S_T AURKA LIKE KINASE), has been chosen for *in silico *spatial structure prediction (Figure 9). Sequence identity and similarity between STALK and Aurora are 60.1% and 81.7%, respectively (Table 2). In addition, identical and similar residues of STALK are distributed evenly along the whole protein chain (Figure 7), resulting in high reliability of the predicted spatial structure.

Differences in spatial orientation between the STALK residues in the active centre (Asp141, Lys143) and the ATP-binding site (Leu24, Gly25, Arg26, Gly27, Lys28, Phe29, Gly30, Arg31, Val32, Lys47) and Aurora A do not exceed 0.3 Å, while the corresponding value for the whole STALK domain is 0.43 Å. This finding confirms more conserved spatial structure of these areas in relationship to the whole protein. Together with the high sequence homology, it further confirms similar properties and catalytic functions of the template and target proteins. The largest difference of the 3D structures between STALK and Aurora is observed in STALK's positions 169-173 (corresponding partly to the activation loop in Aurora A containing pThr288 which is crucial for Aurora A activation [54]) and 233-238 (marked "c" and "d", respectively, on Figure 9). In addition, Thr171 of the STALK's first variable range is identical to Thr288 of Aurora (Figure 9).

**Figure 10:**
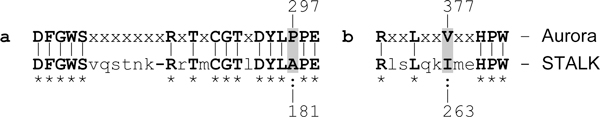
**Conserved motifs typical for Aurora animal kinases found in STALK protein from *V. vinifera*: a - activation loop conserved motif and b - D2-type destruction box**. Marked are the residues different in both proteins.

The activation loop of animal Aurora kinases contains a conserved motif (DFGWSxxxxxxxRxTxCGTxDYLPPE) carrying Thr288 phosphorylated by the cAMP-dependent kinase (Figure 10) [54]. Also, the C-terminal part of the Aurora kinases catalytic domain contains a so-called D2-type destruction box motif [Rxx(L/I)xxVxxHPW] which is thought to play a role in proteasome-dependent protein degradation [53].

In the STALK sequence we have identified, with near 100% identity, both motifs essential for animal Auroras (Figure 10). This confirms functional homology of the STALK (A7PY12/GSVIVT00026259001) protein and human STK6 kinase.

Thus, the results of the search for plant homologues of human kinases SLK, PAK6, PAK7 MARK1, MAST2, TTBK1, TTBK2, AURKA, PLK1, PLK4 and PASK clearly demonstrated the existence of corresponding plant kinases SLK, MAST2 and AURKA. Plants do not seem to contain homologues of kinases PAK6, PAK7, MARK1, TTBK1, TTBK2, PLK1, PLK4 and PASK. This observation could be explained by significant differences in microtubule proteins between plants and animals. Despite the high similarity of animal and plant tubulins and the presence of plant homologues for some other proteins like MAP1 [56], there are differences in a number of microtubule proteins. Plants, for example, do not possess the type of MAP that contains tau repeats (MAP2) [16,56], which explains absence of some protein kinases phosphorylating given substrates in plants. At the same time, kinases involved in pathways common in animal and plant cells and phosphorylated conserved sites in microtubule proteins (tubulin, MAP1, motor proteins, etc.), exhibit considerable similarity.

**Figure 11:**
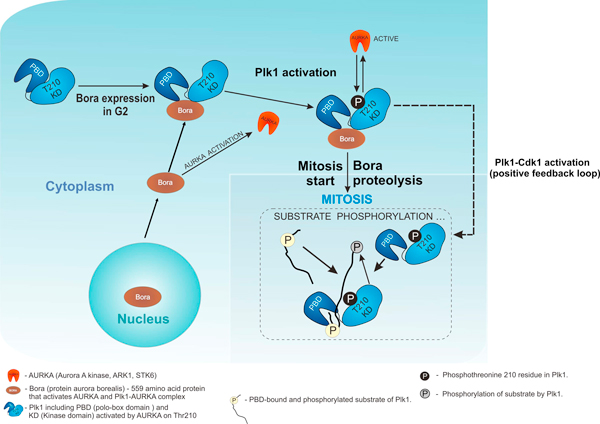
**Interplay of Plk1, AURKA and BORA in cell cycle based on literature data: adapted from **[43,46]**and is based on references **[29,38,43,44,46,57].

An example of the corresponding homologue is Aurora A, a kinase responsible for daughter centrosome formation, asymmetric cell division and mitotic spindle formation [46]. It is believed that the signal from the cyclin-dependent kinase Cdk1 is relayed to the "second" level kinases, one of which is Aurora A [38,46]. In human and animal cells alike, AURKA (Aurora kinase A) is involved in the initiation of mitosis [30]. Activation of AURKA occurs by means of AURKA - BORA (protein BORA, FLJ22624; RP11-342J4.2; C13orf34) complex formation [29,38,43,57]. A mechanism was suggested recently, according to which BORA and AURKA together control transition from phase G2 of cell cycle to phase M [44]. According to the proposed mechanism, Cdk1 causes BORA to exit from the nucleus and binds reversibly with Plk1. This changes Plk1 conformation so that its Thr210 in the catalytic domain becomes accessible for phosphorylation by AURKA with subsequent Plk1 activation [44]. This leads to further Cdk1 activation, degradation of Bora and, eventually, to start of mitosis [44,46] (Figure 11).

STALK, the closest plant homologue for human Aurora kinase, may have a similar mode of interaction in plant cells.

In relation to the potential plant homologues of kinase SLK, some questions remain. Human SLK kinase is highly expressed in various cell types and is involved in the regulation of microtubule radial orientation [44]. By analogy to the *Xenopus laevis *polo-like kinase xPlk1 [58], it may be hypothesized that human protein kinase SLK regulates activity of Plk1, the homologue of the *X. laevis *Plx1 [32]. However, so far the data is inconclusive.

In summary, we have identified plant homologues of human kinases SLK, MAST2 and Aurora A. MAST2 plant homologues are identified here for the first time. BLASTp scanning did not produce any plant homologues of human PAK6, PAK7, MARK1, TTBK1, TTBK2, PLK1, PLK4 and PASK. We hope that new bioinformatic and instrumental genomic and proteomic data will reveal additional plant kinases involved in microtubule protein phosphorylation and cell division regulation.

## Methods

Target sequences of human protein kinases SLK, PAK7, PAK6, MARK1, MAST2, TTBK1, TTBK2, AURKA, PLK1, PLK4 and PASK were obtained from UniProtKB http://www.uniprot.org/ and NCBI GenBank http://www.ncbi.nlm.nih.gov/Genbank/[59] based on the information presented in respective publications. The target sequences were used to search for plant homologues against the UniProt database (Swiss-Prot and TrEMBL) [60]. We performed a BLASTp query (SIB BLAST Network Service) with the protein weight matrix BLOSUM62, with the following settings: (the E threshold = 10 (number of expected matches in a random database), "Filter the sequence for low-complexity regions" and "Gapped alignment" http://www.clustal.org[21].

The domain organization of identified plant homologues was analysed with Simple Modular Architecture Research Tool (SMART - http://smart.embl-heidelberg.de/) [50], Pfam http://pfam.sanger.ac.uk/[61], PROSITE http://www.expasy.ch/prosite/[62] and data from the Swiss-Prot database. Protein kinase relation to specific kinase family was confirmed on the basis of SMART scanning results. Anonymous SMART scanning (RAW-format) was performed with activation of all analytical modules. We also took into account the results of pairwise alignments of query and subject sequences as well as the results of the SMART analysis regarding domain composition and organization (including HMMER results). Homologues were selected based on the meaning of the sequence identities (%), positives % and E-value [63]. Multiple amino acid alignments were generated with ClustalX (2.0.5) http://www.clustal.org using the BLOSUM protein weight matrix [64].

Phylogenetic trees of plant homologues and human SLK, MAST2 and STK6 (AURKA, Aurora A) were generated based on multiple alignment results for the protein kinase domain (SLK, MAST2) [19,20] or the full sequence (STK6), using the neighbour-joining (NJ) algorithm [65,66]. The boundaries of the protein kinase domain were determined using SMART profiles. Dendrogram analysis and visualization was performed using the TreeView X Ver.5 http://taxonomy.zoology.gla.ac.uk/rod/treeview.html[67] and MEGA http://www.megasoftware.net/[68] programs. Conserved functionally important residues and motifs in plant homologues were determined based on database annotation for template sequences of corresponding human protein kinases http://www.expasy.org.

For the three-dimensional (3D) structure reconstruction of A7PY12/GSVIVT00026259001 from *V. vinifera *we used method homologous (profile) modelling [69]. As a matrix we used a 3D structure of AURORA2 kinase in a complex with the inhibitor PHA-6806260 [70] from the Protein Data Bank (PDB) (PDB ID 2J4Z) [71]. Pro-model geometry optimisation was performed by energy minimization using the L-BFGS method [72]. Visualization of obtained three-dimensional model was done using program DS Visualizer 2.0 (Accelrys Software Inc. - http://accelrys.com/). Differences between structures of the STALK and AURORA were estimated by calculating RMSD (root median square deviation) using the Swiss-Pdb Viewer 4.0 software [73].

## Competing interests

The authors declare that they have no competing interests.

## Authors' contributions

PAK made a major contribution to conception and design, acquisition of data, analysis and interpretation of bioinformatic data: blast query, analysis of domain organization, alignments and sequences analysis, cladistic analysis. ESN was involved in revising the manuscript critically for important intellectual content. AIY took part in discussion and interpretation of data (blast query, sequence alignments, cladistic data). VGM made contributions to conception and design, acquisition of data, analysis and interpretation of data. AYN performed acquisition of spatial structure data, analysis and interpretation, molecular modelling and was involved in drafting of the part of manuscript dedicated to spatial structure analysis. NYS participated in the discussion of sequence alignments data. YBB took part in discussion of data, contribution to conception, design and interpretation of data and coordination of whole project. All authors read and approved the final manuscript.
